# A Bayesian Framework for the Network Analysis of Transmission Dynamics in Infectious Disease

**DOI:** 10.1007/s00239-026-10303-w

**Published:** 2026-02-18

**Authors:** Jianing Xu, Jihyun Kim, Pengsheng Ji, Lili Yu, Christopher C. Whalen, Liang Liu

**Affiliations:** 1 Department of Statistics, University of Georgia, 310 Herty Drive, Athens, GA 30606 USA; 2 Department of Biostatistics, Epidemiology and Environmental Health Sciences, Jiann-Ping Hsu College of Public Health, Georgia Southern University, Statesboro, GA 30460 USA; 3 Global Health Institute, College of Public Health, University of Georgia, Athens, GA 30606 USA; 4 Institute of Bioinformatics, University of Georgia, Athens, GA 30602 USA

**Keywords:** Transmission network, Infectious disease, Bayesian estimation, Network structural correlation

## Abstract

Understanding the transmission dynamics of infectious diseases is critical for effective public health intervention. Traditional models often rely on simplifying assumptions that overlook the complexity of real-world contact patterns. In this study, we present an extended Bayesian framework that integrates genomic, temporal, and network data to reconstruct transmission networks with greater accuracy. By incorporating network structure as a prior, the model accounts for social and spatial proximity, allowing transmission probabilities to vary with contact or social distance. We further enhance inference sensitivity through a hypothesis testing procedure optimized via constrained likelihood estimation. Simulation results demonstrate that network-informed models outperform non-network-informed models, particularly under limited genetic resolution. Application to a tuberculosis dataset from Kampala, Uganda reveals that the network-informed model resolves transmission ambiguities more effectively than models based solely on genetic and temporal data. Additionally, Exponential Random Graph Model (ERGM) analysis indicates that transmission is more likely to occur through weak social ties than within tightly connected clusters, aligning with sociological theories of information flow. While the framework shows strong performance, limitations such as data sparsity and computational demands remain. Future work will focus on integrating mobility data to further refine transmission inference. This integrative approach offers a robust tool for epidemiological analysis and supports more targeted public health decision-making.

**Supplementary Information:**

The online version contains supplementary material available at 10.1007/s00239-026-10303-w.

## Introduction

Understanding the dynamics of infectious disease transmission is central to epidemiology, informing both theoretical advances and practical applications in surveillance, intervention, and policy (Anderson and May 1991; Diekmann et al. 2013). Direct transmission processes, such as person-to-person or airborne transmission, emerge from the interplay of biological determinants, such as pathogen characteristics and host immunity, with behavioral and social mechanisms that structure patterns of contact (Eames and Keeling 2002; Funk et al. 2009). Classical compartmental models, particularly the Susceptible–Infectious–Recovered (SIR) framework, have provided the conceptual foundation for modern infectious disease epidemiology (Kermack and McKendrick 1939; Hethcote 2000). By partitioning a population into discrete epidemiological states, the SIR model has enabled rigorous analysis of epidemic thresholds, the basic reproduction number (R0), and the projected effectiveness of control measures, including vaccination and quarantine strategies (Barbour et al. 1996; Anderson and May 1991). Despite their historical importance and continued utility, these mean-field compartmental models rely on simplifying assumptions that limit their capacity to capture the complexities of real-world transmission. Foremost among these is the assumption of homogeneous mixing, whereby all individuals are equally likely to interact. Such an assumption disregards the heterogeneity of contact patterns and the structural constraints imposed by social, demographic, and spatial networks (Keeling and Eames 2005; Bansal et al. 2007). Empirical evidence consistently demonstrates that transmission risk is unevenly distributed, concentrated within specific subgroups or shaped by community-level structures (Mossong et al. 2008; Salathé and Jones 2010). Consequently, models based solely on population averages may obscure critical features of epidemic dynamics, underestimating the role of heterogeneity and network-mediated processes in shaping outbreak trajectories (Pastor-Satorras et al. 2015; Danon et al. 2013).

Recent advancements in network science and computational epidemiology have underscored the critical role of individual-level interactions in shaping infectious disease dynamics (Ganesh et al. 2005; McKee and Dallas 2024; Miller et al. 2021a). Networks offer a robust framework to represent diverse forms of connectivity, including social ties (e.g., friendships, family relationships), contact patterns (e.g., interactions in workplaces or schools), and spatial mobility (e.g., commuting or travel behaviors) (Toole et al. 2015). Key network properties, such as node degree distribution, clustering, and centrality, can profoundly influence transmission outcomes and modulate the effectiveness of interventions, including social distancing or targeted vaccination (Salathe et al. 2010; Green and Kiss 2010; Wallinga et al. 1999). Integrating network structures into epidemiological models offers a more nuanced understanding of disease spread than traditional compartmental approaches alone (Mao and Yang 2012). Such a multi-layered approach provides a comprehensive framework for understanding complex transmission dynamics, ultimately supporting more effective public health decision-making (Sahneh et al. 2019; Nasir 2023).

The integration of genomic data has revolutionized our ability to trace transmission pathways (Pearson et al. 2025; Gay et al. 2024; Sun et al. 2024; Abdullahi et al. 2024). High-throughput sequencing enables the reconstruction of phylogenetic relationships among pathogen strains, offering insights into evolutionary history and transmission events. Genetic markers such as single nucleotide polymorphisms (SNPs) are particularly valuable for identifying transmission pathways and understanding how pathogens evolve over time (Van der Roest et al. 2025; Dawson et al. 2021). By mapping genetic relatedness onto the contact/social networks established from epidemiological data, researchers can visualize how infections spread between individuals, creating a clearer picture of transmission dynamics. Traditional phylogenetic approaches typically focus on genetic relationships between strains, creating trees that depict the evolutionary history of pathogens (Didelot et al. 2021; Bouckaert et al. 2019; Volz et al. 2009). Combining genomic, temporal, and network data provides a more comprehensive view of how infections propagate (Xu et al. 2025). However, current methods for estimating transmission dynamics often fall short of fully integrating the underlying network structures into phylogenetic models.

This study introduces a Bayesian framework that integrates network theory with probabilistic modeling to infer transmission dynamics of directly transmitted pathogens. Within this framework, genetic and temporal data are incorporated into the likelihood function, while network information serves as the prior to model individual contact probabilities. By integrating genomic, temporal, and network information, the approach can enhance the accuracy of transmission network reconstruction and support more effective public health decision-making. The significance of this study lies in its potential to enhance our understanding of complex epidemiological puzzles. By leveraging advanced statistical techniques and network models, we can better predict disease dynamics, evaluate intervention strategies, and ultimately inform policies that are more responsive to the realities of infectious disease transmission. As we confront increasingly complex health challenges in a densely interconnected world, this integrative approach promises to improve our preparedness and response to infectious disease outbreaks.

## Materials and methods

### Bayesian Transmission Model with a Network-Based Prior

In our prior work, we developed a Bayesian transmission model to infer infection dynamics by integrating temporal and genomic data (Xu et al. 2025). The temporal data comprises the symptom onset times $\:{T}^{O}=({T}_{1}^{O},\dots\:,\:{T}_{n}^{O})$ and removal times $\:{T}^{R}=({T}_{1}^{R},\dots\:,\:{T}_{n}^{R})$ for $\:n$ infected individuals, where removal is defined as the time at which an individual ceases to be infectious due to treatment or death. The latent period $\:{t}_{i}^{L}$ of individual $\:i$ is defined as $\:{t}_{i}^{L}={T}_{i}^{O}-{t}_{i}^{I}$ where $\:{t}_{i}^{I}$ represents the unobserved infection time of individual $\:i$. The alignment of $\:n$ pathogen genomes is represented by $\:D=({D}_{1},\dots\:,{D}_{n}),$ where $\:{D}_{i}$​ is the nucleotide sequence for the pathogen genome from individual *i*. The transmission network $\:{\Phi\:}$ is modeled as a directed tree comprising $\:n$ nodes (infected individuals) and $\:(n-1)$ edges $\:\left({\varphi\:}_{2},\dots\:,{\varphi\:}_{n}\right)$, where each edge $\:{\varphi\:}_{i}$ identifies the infector of individual ; individual 1 is treated as the index case and therefore has no infector. These $\:n-1$ edges may correspond to either direct or indirect transmissions. Direct transmission, such as person-to-person contact or airborne spread, occurs through close physical proximity and interactions between hosts, enabling pathogens to transfer via respiratory droplets, aerosols, or direct contact. In contrast, indirect transmission refers to a transmission event occurring through one or more unsampled or unobserved intermediate hosts rather than directly between two observed cases. Such events typically arise when the complete set of infected individuals is not available, leaving gaps in the observed transmission chain. The Bayesian model is parameterized by the transmission tree $\:{\Phi\:}$, the latent periods $\:{t}^{L}$, within-host effective population size $\:\theta\:$ governing the coalescent dynamics within infected hosts and along transmission branches, infection rate $\:\alpha\:$, removal rate $\:\beta\:$, and mutation rate $\:\mu\:$. The joint posterior of model parameters given the temporal and genomic data, enables inference of transmission dynamics and evolutionary history, i.e.,

1 $$ \begin{aligned} & P\left( {\Phi \:,\:t^{L} ,\:\theta \:,\:\alpha \:,\:\beta \:,\:\mu \:|D,\:T^{O} ,\:T^{R} } \right) \propto \:P\left( {D,\:T^{O} ,\:T^{R} |\Phi \:,\:t^{L} ,\:\theta \:,\:\alpha \:,\:\beta \:,\:\mu \:} \right) \\ & \; \times \:P\left( {t^{L} ,\:\theta \:,\:\alpha \:,\:\beta \:,\:\mu \:} \right) \times \:P\left( {\Phi \:} \right) \\ \end{aligned} $$

In (1), $\:P\left(D,\:{T}^{O},\:{T}^{R}|{\Phi\:},\:{t}^{L},\:\theta\:,\:\alpha\:,\:\beta\:,\:\mu\:\right)$ is the likelihood function; $\:P\left({t}^{L},\:\theta\:,\:\alpha\:,\:\beta\:,\:\mu\:\right)$ is the prior of the model parameters $\:\left({t}^{L},\:\theta\:,\:\alpha\:,\:\beta\:,\:\mu\:\right)$; $\:P\left({\Phi\:}\right)$ is the prior of the transmission events assuming a constant transmission probability between any two individuals. In this paper, the Bayesian framework is further extended by incorporating a predefined network $\:G$ that accounts for heterogeneous contact structures. In network $\:G$,each edge represents a direct social/contact/mobility connection and has unit length; given this, the geodesic distance between any two nodes corresponds to the minimum number of edges that must be traversed to connect them. The network $\:G$ is integrated into the Bayesian model as a hyperparameter, modifying the prior probability $\:P\left({\Phi\:}\right)$ of transmission events in the transmission tree $\:{\Phi\:}$. In the previous work, transmission events among different transmitters are assumed to follow the uniform distribution, i.e., all possible transmissions are equally likely. In the extended model, the prior probability of transmission events depends on the network $\:G$, i.e., $\:P\left({\Phi\:}\right|G)\:$, allowing the model to integrate social or spatial proximity in estimating transmission probabilities. With the assumption of independence among transmission events, the prior probability $\:P\left({\Phi\:}\right|\mathrm{G})$ of the transmission tree $\:{\Phi\:}$ can be expressed as the product of $\:n-1$ independent transmission events $\:({\varphi\:}_{2},\dots\:,{\varphi\:}_{n})$:

2 $$ \:P\left( {\Phi \:|G} \right) = \prod {\:_{{i = 2}}^{n} } P\left( {\varphi \:_{i} |G} \right) $$

The probability $\:P\left({\varphi\:}_{i}\right|G)$ of a transmission event from a transmitter *j* to individual $\:i$ is determined by their geodesic distance $\:{g}_{i,j}$ in the network $\:G$, i.e., the minimum number of edges connecting $\:j$ and $\:i$. We assume that the probability of transmission decays exponentially with distance in the contact network $\:G$, i.e., $\:P\left({\varphi\:}_{i}\right|G)\propto\:\:{e}^{-{g}_{i,j}}$. After normalization across all possible transmitters of $\:i$ (i.e., $\:j=1,\cdots\:,i-1$), the prior probability of a transmission event is given by

3 $$ \:P\left( {\varphi \:_{i} |G} \right) = \:\frac{{e^{{ - g_{{i,j}} }} }}{{\sum {\:_{{j = 1}}^{{i - 1}} } e^{{ - g_{{i,j}} }} }}\:\:\:\:\:for\:j = 1, \ldots \:,i - 1 $$.

The Bayesian estimates of the model parameters $\:({\Phi\:},{t}^{L},\theta\:,\alpha\:,\mu\:)$ are obtained using samples generated from the Metropolis–Hastings algorithm. This Markov Chain Monte Carlo (MCMC) approach iteratively updates the parameters until convergence to the target posterior distribution is achieved. Since the removal rate $\:\beta\:$ is assigned a conjugate prior - an exponential prior with rate $\:\delta\:$, its posterior distribution follows a Gamma distribution with $\:a=n+1$ and $\:b=\delta\:+\sum\:_{i=1}^{n}\left({T}_{i}^{R}-{T}_{i}^{O}\right)$. Therefore, the Bayesian estimate of $\:\beta\:$ is the posterior mean $\:{\widehat{\beta\:}}_{Bayes}=\frac{n+1}{\delta\:+\sum\:_{i=1}^{n}\left({T}_{i}^{R}-{T}_{i}^{O}\right)}$.

### Hypothesis Testing of Direct Transmissions Using L-BFGS-B Optimization

The edges in an inferred transmission network may represent either direct or indirect transmissions due to incomplete case sampling. Accurately inferring direct transmissions is critical because they reveal the true pathways of pathogen spread among observed individuals, allowing for precise reconstruction of transmission networks. Identifying these direct links supports estimation of key epidemiological parameters, such as generation time and reproduction number, and informs targeted interventions by pinpointing high-risk contacts or settings. In our previous work, we introduced a hypothesis testing framework to evaluate whether observed single nucleotide polymorphism (SNP) distances between inferred transmission pairs are consistent with direct transmission expectations. This approach enables systematic evaluation of the plausibility of direct links in partially observed transmission networks. The performance of the test is determined by the estimates of the model parameters $\:\theta\:$ and $\:\mu\:$. Since the parameters $\:\theta\:$ and $\:\mu\:$ are estimated from all transmissions (i.e., indirect and direct transmissions) in the transmission network $\:{\Phi\:}$, the Bayesian estimates of $\:\theta\:$ and $\:\mu\:$ may undermine the power of the test. Here, we employ a limited-memory BFGS algorithm with box constraints (L-BFGS-B) (Byrd et al. 1995) to re-estimate $\:\theta\:$ and $\:\mu\:$ from direct transmission pairs. The algorithm starts with posterior estimates and iteratively minimizes the negative log-likelihood of SNP differences among direct pairs (Supplementary Material S1). Updated parameters refine hypothesis thresholds and reclassify pairs until classifications stabilize (Supplementary Material S1). This approach improves sensitivity of the hypothesis test while maintaining type I error control.

### Network Structural Correlation Analysis with Exponential Random Graph Models

Understanding how transmission events correlate with different types of interpersonal connections is critical for identifying structural drivers of infectious disease spread. In this study, we examined whether transmission likelihood is associated with proximity in a predefined social/contact/mobility network, where edges represent direct social, contact, or mobility ties. Individuals within households or communities typically exhibit shorter network distances, making social proximity a plausible predictor of transmission. To formally assess this relationship, we employed Exponential Random Graph Models (ERGMs) (Hunter et al. 2008; Krivitsky 2012), which provide a statistically rigorous framework for modeling network data while accounting for dependencies among edges. Unlike conventional regression models, ERGMs are designed to capture structural features such as triadic closure, mutual ties, and degree heterogeneity, which are common in social and contact networks. Particularly, ERGMs model the probability of observing a network configuration $\:Y=y$ given covariates $\:X$ as:

4 $$ \:P\left( {Y = y|X} \right) = \frac{1}{{c_{{\phi \:}} }}e^{{\sum {\:_{k} } \phi \:_{k} \:s_{k} (y,X)}} $$

where $\:{s}_{k}(y,X)$ are network statistics (e.g., number of edges, triangles, shared partners), $\:{\phi\:}_{k}$ are model parameters, and $\:{c}_{\phi\:}={\sum\:}_{y}{e}^{{\sum\:}_{k}{\phi\:}_{k}\:{s}_{k}(y,X)}$ is a normalizing constant. In our application, the response variable $\:{Y}_{ij}$ indicates whether a transmission occurred between individuals $\:i$ and $\:j$, while the covariate vector $\:{X}_{ij}$ includes the geodesic distance in the social/contact/mobility network. The covariate vector $\:{X}_{ij}$ can be extended to incorporate additional nodal and dyadic attributes, such as shared group membership or centrality measures (Hunter et al. 2008). Transmission events were inferred using our Bayesian framework and classified as present ($\:{Y}_{ij}=1$) if the posterior probability exceeded a predefined threshold (e.g., 0.5). We fit ERGMs using a logistic pseudolikelihood formulation, in which the conditional log-odds of an edge between and are modeled as

5 $$ \:log\left( {\frac{{p\left( {Y_{{ij}} = 1} \right)}}{{1\: - \:p\left( {Y_{{ij}} = 1} \right)}}} \right)\: = \:\theta \:^{T} X_{{ij}} $$.

This approach allowed us to quantify the influence of network distance and other structural features on transmission probabilities, while controlling for global network dependencies. When applied to social/contact/mobility networks, ERGMs offer a principled method for evaluating whether shorter distances — indicative of more frequent or intimate interactions — correlate with increased transmission risk.

### Simulation

**Fig. 1:**
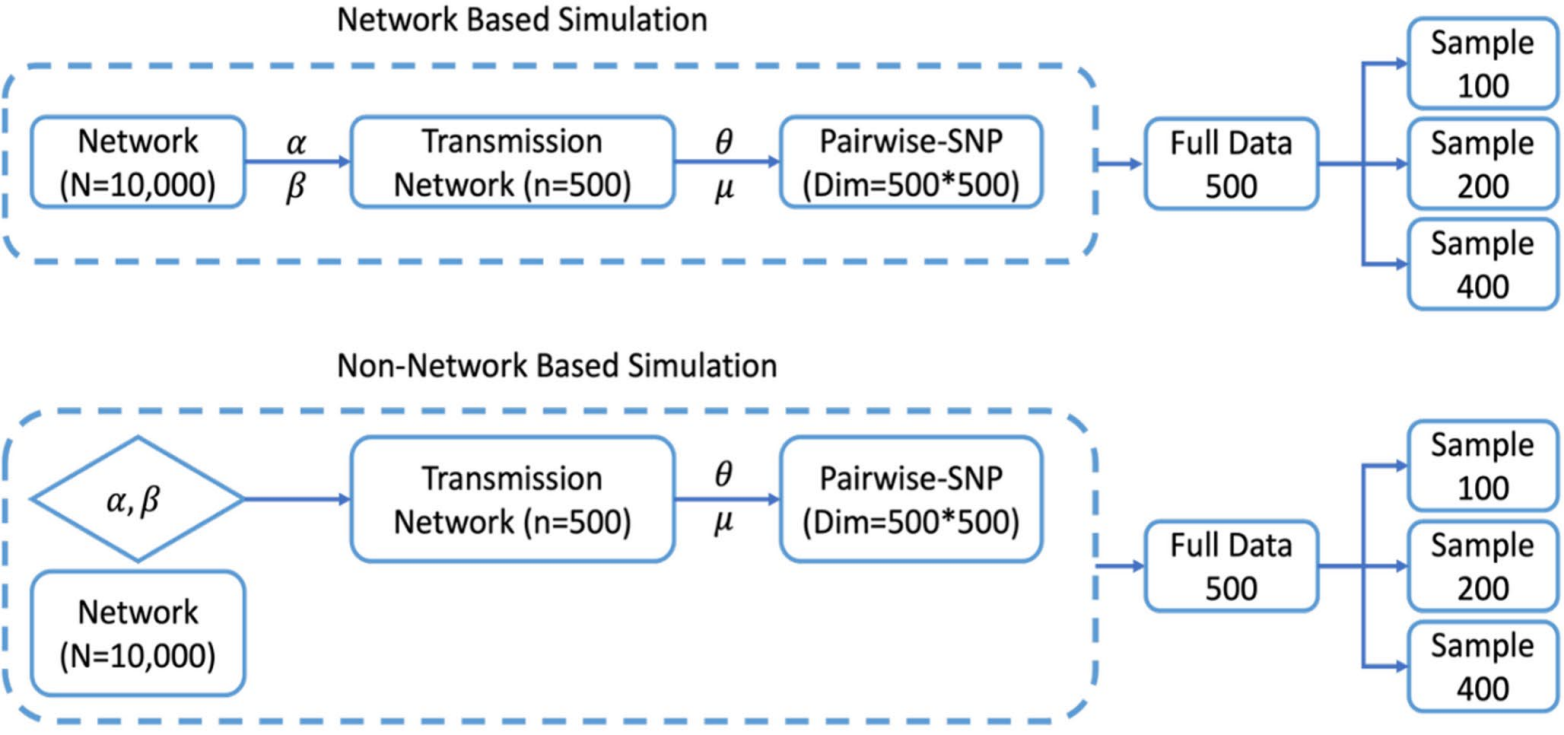
Workflow for network-based and non-network-based simulations. Network-based simulation: A contact network of 10,000 nodes is generated using a hybrid Erdős–Rényi and Barabási–Albert model. Disease spread is simulated over this network until 500 individuals are infected, incorporating geodesic distance as a proxy for contact proximity. Non-network-based simulation: Follows the same procedure without network structure. Both workflows produce transmission networks of 500 cases, pairwise SNP matrices, and subsamples of size 100, 200, and 400 for analysis

To evaluate the performance of our Bayesian framework, we conducted both network-based and non-network-based simulations (Fig. 1 and Supplementary Material S2). The non-network-based simulations follow the same procedure as the network-based ones, except that the network component is omitted. For the network-based simulations, we construct a contact proximity network $\:{\Psi\:}$ of 10,000 nodes using Erdős-Rényi (ER) and Barabási-Albert (BA) models (Erdős and Rényi 1960; Barabási and Albert 1999) implemented in the igraph package in R (Supplementary Material S2). These models were chosen because transmission patterns simulated from ER and BA networks closely resemble those observed in our empirical data analysis. All edges in the network are assigned unit length. Disease spread was simulated by initiating infection in a randomly chosen individual and iteratively propagating transmission events through the network $\:{\Psi\:}$ until 500 infections were reached (Behr, Edelstein, and Ramakrishnan 2018) (Supplementary Material S2). In parallel with the temporal dynamics, we simulated genomic evolution by generating single nucleotide polymorphism (SNP) data. The genome of the initial infection (individual *i* = 1) was constructed under the Jukes–Cantor substitution model (Jukes and Cantor 1969), with nucleotides sampled at equal probabilities. Rather than storing full genome sequences for all individuals, we tracked only the mutation loci and corresponding substitutions relative to this reference genome, allowing efficient simulation and storage. SNP counts for direct and indirect transmission pairs were simulated separately (Supplementary Material S3).

### Performance of the Bayesian Transmission Model With/Without Network Information

Using the simulation framework described above, we first constructed a contact proximity network of 10,000 individuals and then simulated a transmission network of 500 infections by setting this value as the stopping criterion. The spread of infection was governed by specified infection and removal rate $\:\left(\alpha\:,\:\beta\:=1.5,\:2,\:3\right)$ (Supplementary Material S4-5). Unlike models restricted to direct contacts, our framework allowed infection probability to vary with network distance, enabling transmission between indirectly connected individuals based on proximity within the network. Subsequently, we generated pairwise SNP distance matrices using the mutation rate $\:(\mu\:=5\times\:{10}^{-7},1\times\:{10}^{-6},2\times\:{10}^{-6})\:$and within-host effective population size $\:(\theta\:=1\times\:{10}^{-6},2\times\:{10}^{-6},5\times\:{10}^{-6})$ and genome length $\:(N=1\times\:{10}^{6},\:4.4\times\:{10}^{6})$. In total, data were simulated under eight combinations of $\:\alpha\:$, $\:\beta\:$, $\:\mu\:$, and $\:\theta\:$ for $\:N=4.4\times\:{10}^{6}$ (Supplementary Material S4), and six combinations for $\:N=1\times\:{10}^{6}$ (Supplementary Material S5). In the non-network-based simulation, transmission was simulated independently of the contact proximity network, relying solely on infection and removal rates (Xu et al. 2025).

Furthermore, we randomly sampled $\:100\:\left(20\%\right)$, $\:200\:\left(40\%\right)$, and $\:400\:\left(80\%\right)$ of the infected individuals to define subpopulations. For each sample, we reconstructed relevant information, including temporal infection data and pairwise SNP distance matrices. Specifically, temporal data were recalibrated by setting the earliest infection time within the sample as the reference (time $\:0$). Each simulation was repeated three times to ensure robustness. The simulated datasets were analyzed using our Bayesian transmission model. For each scenario, we ran a Markov Chain Monte Carlo (MCMC) algorithm for 100,000 iterations, with the first 20,000 iterations serving as burn-in. After the burn-in phase, parameter estimates were recorded every 100 iterations. Convergence of the MCMC algorithm was assessed using trace plots of the logarithm of posterior probabilities.

To evaluate the accuracy of inferred transmission networks, we focused on two key metrics: overall edge accuracy and direct edge accuracy. Overall edge accuracy measures the proportion of correctly inferred transmission events (edges) in the estimated network, reflecting its topological similarity to the corresponding subtree of the true transmission network. Direct edge accuracy, by contrast, assesses the proportion of correctly inferred edges among direct transmission pairs — those node pairs that are adjacent in the full transmission tree within the sampled subnetwork (100, 200, or 400 individuals). Accuracy values were averaged across three simulation replicates for each sampling scenario to ensure robustness.

### Performance of the Hypothesis Test for Identifying Direct Transmissions

Direct edge accuracy is defined as the proportion of correctly inferred edges among direct transmission pairs which are node pairs that are adjacent in the full transmission tree within the sampled subnetwork (100, 200, or 400 individuals). Like overall edge accuracy, this metric assesses topological agreement, but limits evaluation to a subset of edges representing direct transmission events. Accuracy values were averaged across three simulation replicates for each sampling scenario. We assessed performance using the average type I error and average power across simulation replicates. In this framework, the null hypothesis corresponds to a direct transmission, and the alternative corresponds to an indirect transmission. The type I error quantifies the proportion of direct transmission pairs that were incorrectly classified as indirect while the power measures the proportion of indirect transmission pairs that were correctly identified. Together, these metrics evaluate the hypothesis test’s ability to distinguish between direct and indirect transmission events.

### Performance of ERGM in Capturing the Link between Network Distance and Transmission

To evaluate the robustness of our Exponential Random Graph Model (ERGM) in capturing the relationship between network structure and transmission dynamics, we conducted a series of simulations introducing varying levels of structural noise into the contact network. Specifically, we randomly perturbed 5%, 10%, and 20% of the network ties across three replicates per noise level, resulting in nine perturbed networks (Supplementary Materials S6). For each noise level, node pairs were selected uniformly at random, and ties were either added or removed depending on their current state in the adjacency matrix. After each perturbation, we recomputed shortest path distances for the individuals in the transmission subnetwork and refitted the ERGM for the updated network and corresponding transmission tree using an R package *ergm* (Hunter et al. 2008).

### Real Data Analysis

To evaluate the performance of our Bayesian transmission framework in a real-world setting, we applied it to a tuberculosis (TB) dataset from Kampala, Uganda, originally collected by Kakaire et al. (Kakaire et al. 2021). This dataset included 123 TB cases and 124 frequency-matched controls, recruited through door-to-door surveys and matched by age group, sex, and parish. Whole-genome sequencing (WGS) data spanning 411,532 base pairs, along with detailed temporal and geographic metadata, were obtained for all participants. Of these, 93 TB patients had complete genomic and temporal data and were selected for analysis in the present study. In addition, a comprehensive social network based on reported relations with informants was constructed from the same set of index participants (123 TB cases and 124 controls) using a two-step egocentric sampling approach (Miller et al. 2021b). Index participants first listed their close contacts, who then named their own contacts, resulting in a network of 11,840 individuals. This network was used to derive pairwise social proximity measures among the 93 TB patients.

To analyze transmission dynamics, we applied our Bayesian framework to these 93 cases, integrating genomic, temporal, and network data. Three independent Markov Chain Monte Carlo (MCMC) chains were run for 1,000,000 iterations each, with the first 20,000 iterations discarded as burn-in. Posterior samples from all chains were pooled to estimate model parameters. Two models were compared: one using only genetic and temporal data, and another incorporating social network information. Transmission trees inferred under both models were compared to assess consistency and the effect of network data on transmission tree estimation. Direct and indirect transmissions were identified using a hypothesis test with optimization. To evaluate the role of social structure, we fitted Exponential Random Graph Models (ERGMs) to the inferred transmission networks, using network distance as a covariate for transmission probabilities.

## Results

### Simulation

#### Performance of the Bayesian Transmission Model With/Without Network Information

**Fig. 2:**
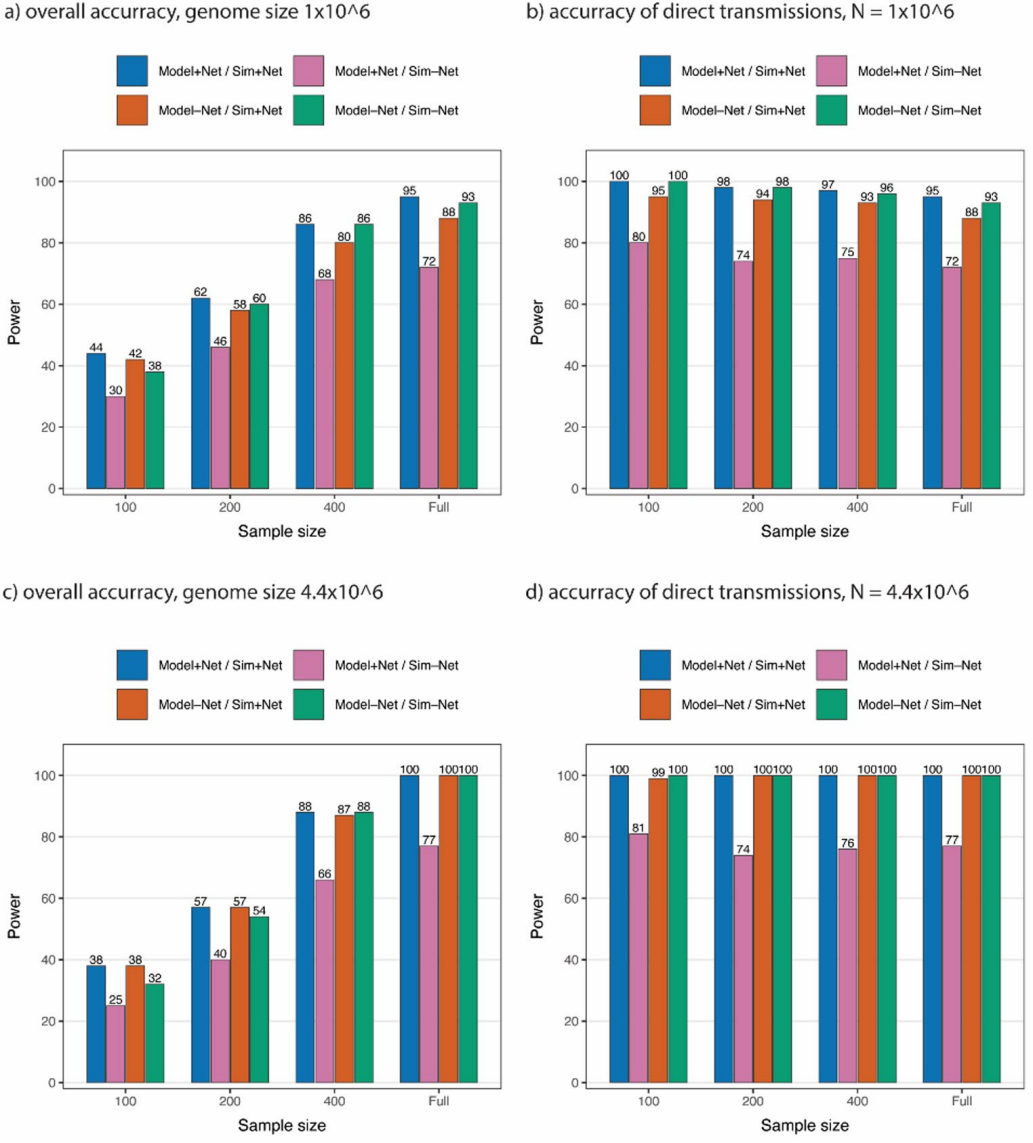
Performance comparison between the network-informed model and the baseline model without network structure. Sim + Net indicates data simulated with network information; Sim-Net indicates data simulated without network information; Model + Net refers to analysis using the network-informed model; Model-Net refers to analysis using the baseline model. **a** Overall Accuracy under different simulation schemes and Bayesian models with genome length $\:1\times\:{10}^{6}$, **b** Accuracy over direct pairs under different simulation schemes and Bayesian models with genome length $\:1\times\:{10}^{6}$, **c** 4 Overall Accuracy under different simulation schemes and Bayesian models with genome length $\:4.4\times\:{10}^{6}$, **d** Accuracy over direct pairs under different simulation schemes and Bayesian models with genome length $\:4.4\times\:{10}^{6}$

To assess the impact of incorporating network data into transmission inference, we compared the performance of the network-informed Bayesian model with a baseline model that excludes network structure. When data were simulated with network information under conditions of limited genetic resolution (genome length $\:N=1\times\:{10}^{6}$), where network structure plays a significant role in transmission, the network-informed model consistently outperformed the baseline model across all sample sizes. In the full sample, it achieved up to a 7% improvement in both metrics, with gains of 2–6% in overall accuracy and 4–5% in direct edge accuracy across subsamples (Fig. 2a-b). As the sequence lengths increased to $\:N=4.4\times\:{10}^{6}$, performance between the two models converged, with overall accuracy ranging from 38% to 100% depending on sample size, and perfect accuracy among direct transmission pairs (Fig. 2c-d). Conversely, in scenarios where transmission was simulated independently of network structure, incorporating network information reduced model performance across both metrics. This highlights the importance of aligning model assumptions with the underlying transmission mechanisms. When network structure is relevant, its inclusion enhances inference; when it is not, it may introduce noise and reduce accuracy.

#### Performance of the Hypothesis Test for Identifying Direct Transmissions

**Fig. 3:**
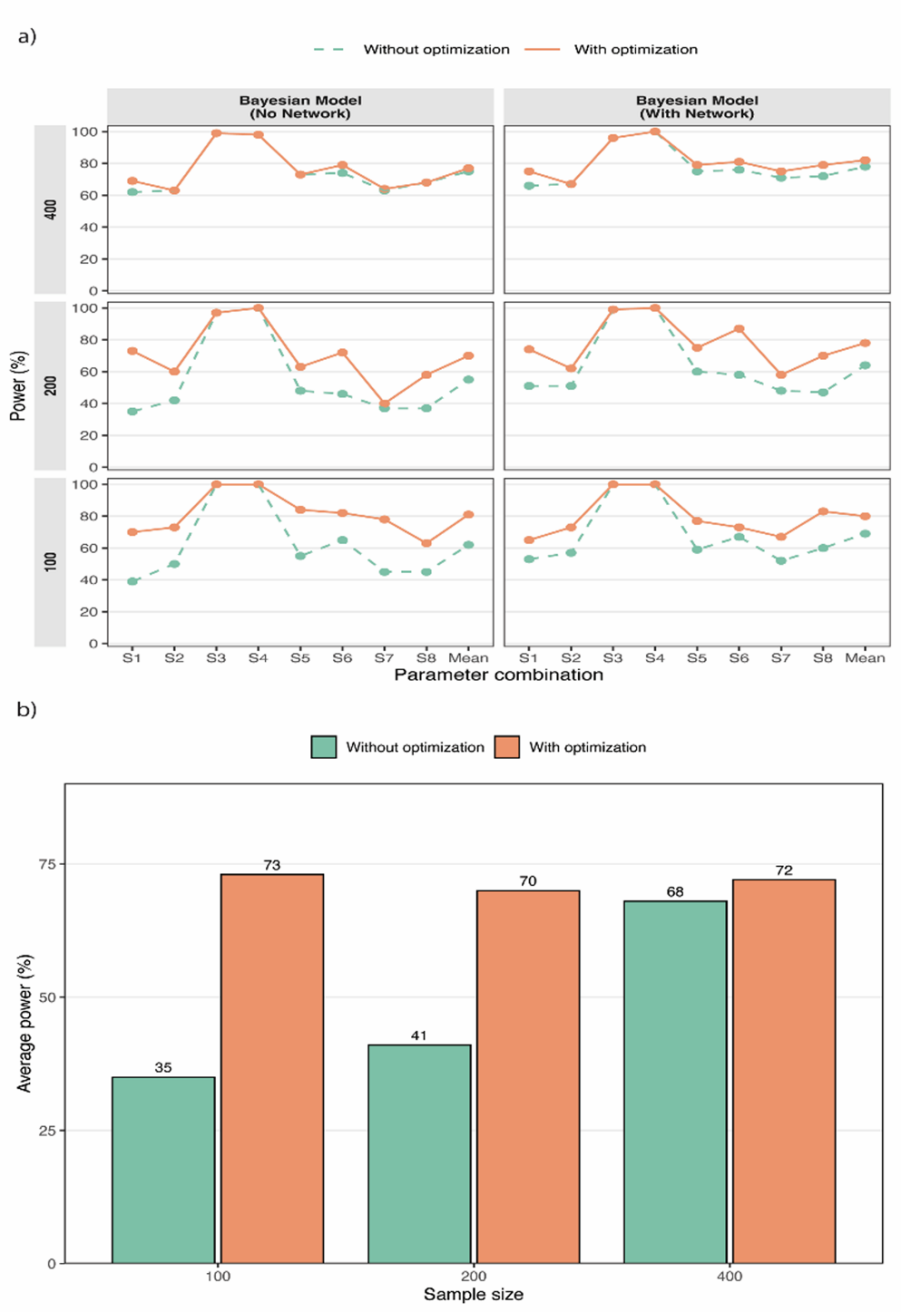
Performance of the hypothesis test for identifying direct transmissions. **a** Power of the hypothesis test under eight parameter configurations S1-S8 (see Supplementary Material S4) plotted along the x-axis and the mean power averaging over eight parameter configurations. **b** Comparison of average power for the hypothesis test with and without optimization across sample sizes of 100, 200, and 400 under parameter setting $\:\theta\:=1\times\:{10}^{-6},\mu\:=2\times\:{10}^{-6},\alpha\:=\beta\:=3$

We compared the performance of the hypothesis test for distinguishing direct from indirect transmission events with and without L-BFGS-B optimization under various simulation scenarios, Bayesian model configurations, and sample sizes. With optimization, substantial gains in power were observed, particularly in smaller sample sizes (Fig. 3a). For instance, in simulations with 100 and 200 individuals, the mean power across simulation schemes increased by 11–19% and 15–20%, respectively, compared to the test without optimization (Fig. 3a). At larger sample sizes (*n* = 400), improvements were more modest, as baseline (without optimization) power was already high. Similar results are observed under parameter setting θ = $\:1\times\:{10}^{-6}$, µ = $\:2\times\:{10}^{-6}$, α = β = 3 (Fig. 3b). At *n* = 400, power increased from 68% to 72%, while at *n* = 200 and *n* = 100, gains were more pronounced, rising from 41% to 70% and from 35% to 73%, respectively (Fig. 3b). These results demonstrate that optimization is most beneficial when data are sparse.

We next examined how the mean power responds to systematic variation in model parameters while holding others constant. When $\:\mu\:$ was fixed at 5$\:\times\:{10}^{-7}$ and $\:\alpha\:=\beta\:=3$, varying $\:\theta\:$ among 1$\:\times\:{10}^{-6}$ (red), $\:2\times\:{10}^{-6}$ (green), and 5$\:\times\:{10}^{-6}$ (blue) produced substantial gains in the mean power, with higher $\:\theta\:$ consistently outperforming lower values (Supplementary Material S7). Holding $\:\theta\:$ = 1$\:\times\:{10}^{-6}$ and $\:\alpha\:=\beta\:=3$, increasing $\:\mu\:$ from 5$\:\times\:{10}^{-7}$ to 2$\:\times\:{10}^{-6}$ yielded monotonic gains. Finally, when $\:\theta\:=$1$\:\times\:{10}^{-6}$ and $\:\mu\:$ at 5$\:\times\:{10}^{-7}$ were fixed, varying $\:(\alpha\:,\beta\:)$ among $\:(3,\:3)$, $\:(2,\:2)$, and $\:(1.5,\:1.5)$ produced only modest changes in the mean power within each sample size, with differences small relative to those seen for $\:\theta\:$ or $\:\mu\:$ (Supplementary Material S7). Overall, the mean power was most strongly driven by sample size, with $\:\theta\:$ and $\:\mu\:$ exerting secondary but substantial effects, and $\:(\alpha\:,\beta\:)$ changes having only minor influence within the ranges tested. These results suggest that maximizing genetic resolution (via higher $\:\mu\:$) or within-host effective population size (via higher $\:\theta\:$) can improve classification accuracy, but adequate sample size remains the most critical factor for reliable inference. In contrast, high infection and removal rates may modestly reduce the mean power, particularly when data are sparse.

Beyond power gains, it is also important to provide practical guidance on the choice of the tuning parameter $\:\lambda\:$. We expressed sample sizes as fractions of the full cohort (500 individuals): 20% (*n* = 100), 40% (*n* = 200), and 80% (*n* = 400). The optimal tuning parameter λ increased with decreasing sample size, ranging from 0.1 to 0.2 at 80% (*n* = 400) to $\:0.3-0.5$ at 40% (*n* = 200) and to 0.4–0.6 at 20% (*n* = 100), indicating that wider bounds are necessary under limited data to maintain power while controlling type I error. These findings highlight the effectiveness of the optimization procedure in enhancing the sensitivity of transmission inference, particularly in small-sample settings, while preserving statistical rigor through controlled error rates.

#### Performance of ERGM in Capturing the Link between Network Distance and Transmission

In the baseline (unperturbed) network, the ERGM included social distance in the social network as an edge-level covariate. The model yielded a significantly negative edge coefficient (− 4.8793), indicating a low baseline probability of transmission links, and a negative coefficient for social distance (− 0.0684), suggesting that each unit increase in network distance reduced the likelihood of transmission by approximately 6.6%. As structural noise increased, the significance of the social distance covariate diminished as expected (Table 1). At 5% perturbation, the standard error remained low (SE = 0.050), and the covariate retained statistical significance. At 10% noise, the standard error increased (SE = 0.068), and p-values varied, with some exceeding 0.3, indicating reduced evidence for a meaningful effect (Table 1). At 20% noise, the standard error rose further (SE = 0.079), and p-values frequently exceeded 0.99, suggesting that the model could no longer detect a significant relationship between network distance and transmission (Table 1). These results demonstrate that the ERGM can detect the true relationship between transmissions and social/contact distances in the underlying network. As noise increases, the model appropriately reflects the decline in the significance of using social/contact distances to predict transmission events. This responsiveness highlights the ERGM’s utility in detecting the true relationship between transmission events and social/contact distances in the social/contact network.

**Table 1:** ERGM results for different levels of network noise (5%, 10%, and 20%). Each noise level is repeated three times

Metric	5% Noise			10% Noise			20% Noise		
	Run 1	Run2	Run3	Run 1	Run 2	Run 3	Run 1	Run 2	Run 3
Estimate	− 0.9	− 0.15	− 0.10	− 0.15	− 0.12	− 0.07	− 0.13	− 0.14	− 0.001
SE	0.05	0.05	0.05	0.068	0.068	0.079	0.079	0.079	0.079
p-value	0.076	0.004	0.057	0.025	0.085	0.164	0.164	0.065	0.994
BIC	6527	6522	6526	6525	6527	6527	6527	6527	6530

#### Real Data Analysis

The study tracked 93 tuberculosis (TB) patients over a 4.4-year period, from initial infection to final removal. Infectious periods varied widely, ranging from 0.08 to 2.08 years, with a median duration of 0.25 years (approximately 91 days) and a mean of 0.37 years. This distribution was right-skewed, driven by a small number of prolonged cases, including two patients with infectious durations exceeding two years. Genomic data from the 93 pathogen strains revealed pairwise single nucleotide polymorphism (SNP) counts ranging from 0 to 1,935, with a median of 662 and a mean of 731. The interquartile range (333–1,167) also indicated moderate right skew, reflecting a subset of genetically divergent cases. The sociometric network of $\:\mathrm{11,840}$ individuals contained a giant component covering $\:84\mathrm{\%}$ of the population and $\:46$ smaller disconnected groups. The probability of a direct connection was just $\:0.02\mathrm{\%}$, and $\:29\mathrm{\%}$ of all pairs were disconnected. Among the $\:93$ TB cases, we computed shortest paths for all $\:\mathrm{4,278}$ unique pairs. $\:51\mathrm{\%}$ of pairs were disconnected, and only $\:3\mathrm{\%}$ had a path length of $\:1$ to $\:5$, indicating limited close connectivity among patients within the broader network.

**Fig. 4:**
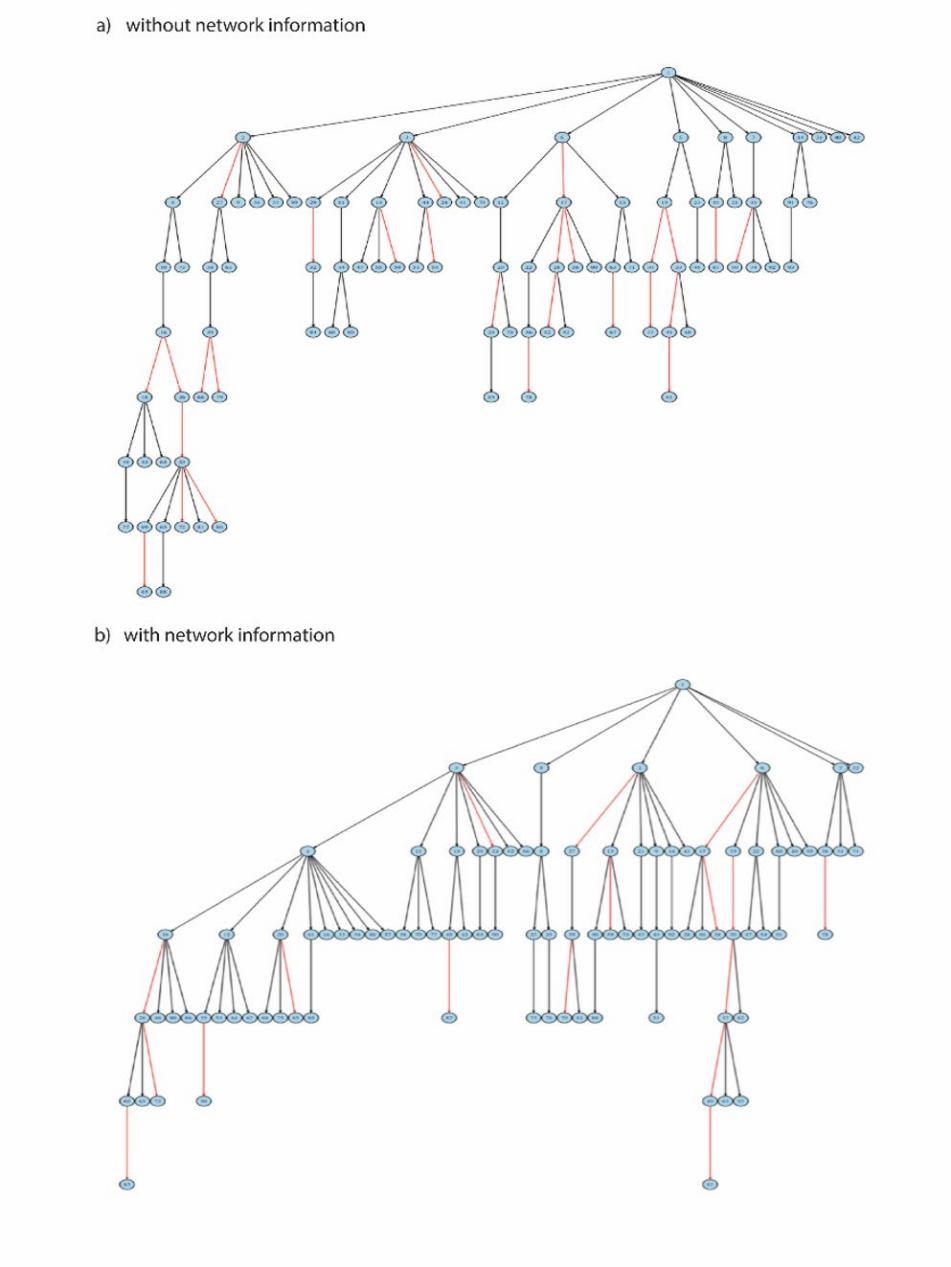
Inferred Transmission Tree for the real data of 93 cases. **a** The transmission tree was estimated by the Bayesian model without network information. Pairs with red edges are inferred as direct transmission and the ones with black are inferred as indirect. **b** The transmission tree was estimated by the Bayesian model with network information. Pairs with red edges are inferred as direct transmission and the ones with black are inferred as indirect

The Bayesian models with and without network data (i.e., pairwise distances in the social network) were applied to the dataset of 93 TB cases. Both models produced similar estimates of the infection rate $\:\alpha\:$ (Supplementary Material S8). In contrast, the network-informed model yielded a higher mutation rate $\:\mu\:$ and a lower within-host effective population size $\:\theta\:$. Moreover, we compared transmission trees inferred from two models (Fig. 4). Across three independent MCMC runs, the model without network information consistently identified 27 direct transmission pairs (Supplementary Material S9), while the network-informed model inferred 17 (Supplementary Material S10). Thirteen pairs were consistently recovered by both approaches and supported by hypothesis testing, highlighting a core set of plausible transmission events. We next applied an ERGM to both inferred transmission networks to evaluate whether social network distance influences tie formation. Distance was included as a covariate, with coefficients interpreted as additive contributions to the log-odds of a tie. In both models, the edges term was negative and highly significant (Supplementary Material S11–S12), reflecting the inherent sparsity of the transmission network. By contrast, the coefficient for distance was positive but not statistically significant (*p* = 0.313 and *p* = 0.867), indicating no meaningful association between shorter social distance and transmission. This is consistent with the descriptive finding that only one direct transmission pair in each network had a distance of two or less, suggesting minimal neighborhood-level spread. Model fit, as indicated by reduced deviance and acceptable AIC (891.4 and 892.4) and BIC (904.2 and 905.2) values, confirmed that the ERGM captured key structural features. However, the weak explanatory role of distance implies that additional covariates may be required to better account for the mechanisms driving transmission ties.

## Discussion

Accurately reconstructing transmission networks is essential for identifying key epidemiological drivers and informing targeted control strategies for directly transmitted infectious diseases. In this study, we extended our previous Bayesian framework by integrating genomic, temporal, and network data to improve the reconstruction of transmission networks for infectious disease. Traditional transmission models that rely exclusively on genetic and temporal data often fail to account for the structural constraints imposed by underlying social or spatial contact networks, resulting in incomplete or ambiguous reconstructions of transmission pathways. In empirical settings, genetic similarity and temporal proximity alone are frequently insufficient to distinguish among multiple plausible infectors, particularly for pathogens with low mutation rates or long latency periods. Incorporating network information as a prior directly addresses this limitation by restricting transmission pathways to those that are epidemiologically plausible given observed patterns of social interaction or spatial proximity. By allowing infection probabilities to decay with network distance, the proposed framework accommodates heterogeneity in contact opportunities and explicitly encodes realistic constraints on who can infect whom. The incorporation of network structure does not merely improve model fit but also enhances interpretability in real-world analyses. When competing transmission hypotheses are supported by similar genetic and temporal evidence, the network prior provides a principled mechanism for resolving ambiguity by favoring transmission pathways that align with observed contact structures. This is particularly valuable in partially observed or sparsely connected networks, where direct contacts are rare and plausible transmission may occur through indirect or weak ties. In such settings, network-informed priors mitigate over-reliance on genetic distance thresholds and reduce the risk of inferring biologically implausible transmission links driven solely by stochastic genetic similarity.

Our simulation analyses highlight an important caveat that when observed networks are noisy, incomplete, or only weakly related to the true transmission process, reliance on network information can attenuate performance. This underscores the need for careful construction and validation of network information in applied settings, as well as sensitivity analyses that assess robustness to network misspecification. At the same time, extensive epidemiological evidence indicates that for infectious diseases transmitted through person-to-person contact, transmission events are inherently structured by social and spatial proximity. Although empirical contact networks are often imperfect representations of true interactions, both epidemiological theory and our empirical findings demonstrate that they nonetheless remain highly informative for modeling transmission dynamics. In the analysis of 93 TB cases, the network-informed and non-network-informed models produced different transmission inferences from the same dataset. Incorporating network information not only altered the inferred transmission pathways but also influenced key parameter estimates, yielding a higher mutation rate (µ) and a lower within-host effective population size (θ). A lower θ suggests reduced genetic variation within hosts, while a higher µ reflects greater divergence among sequences involved in transmission events. In contrast, models without network data tend to prioritize pairs with minimal genetic differences, potentially misrepresenting true transmission routes. For instance, the non-network-informed model identified case 16 as the infector of case 26 (SNP distance = 2), whereas the network-informed model favored case 18 (SNP distance = 4), due to a direct network connection absent between 16 and 26 (Supplementary Material S13). A similar discrepancy was observed for case 49, where the genetic-temporal model selected case 33 (SNP = 0), while the network-informed model inferred case 37 (SNP = 1), supported by a finite network path (distance = 10) between 37 and 49 (Supplementary Material S13). These examples highlight the importance of incorporating contact networks as informative data resources of transmission pathways. As increasingly rich empirical network data become available, from social surveys, mobility tracking, or digital contact tracing, network-informed Bayesian frameworks offer a scalable and transparent approach for integrating heterogeneous data streams into transmission inference.

ERGM analysis of the 93 tuberculosis (TB) cases indicates that transmission events were more likely to occur through weak social ties, casual or infrequent contacts between individuals who are not embedded in the same close-knit social circles, rather than within densely connected clusters. This pattern is consistent with Granovetter’s weak-tie theory (Granovetter 1973), which characterizes weak ties as structural bridges linking otherwise disconnected groups and facilitating the diffusion of information across broader networks. In the case of TB, these bridging connections enable transmission to extend beyond households or tightly connected contact groups, promoting spread into new social and geographic communities. The prominence of weak ties in transmission has important implications for public health practice. Interventions that focus primarily on strong ties, such as household-based contact tracing, may fail to detect a substantial fraction of transmission events that occur through casual or transient interactions (Granovetter 1973). Network-informed strategies that explicitly identify and target bridging nodes may therefore be more effective. From a modeling perspective, these findings highlight the limitations of approaches that rely exclusively on observed interpersonal social ties. A more comprehensive framework could explicitly incorporate environmental or location-based contact structures, yielding multilayer or bipartite network representations that link individuals through shared places such as workplaces, markets, or transportation hubs. Jointly modeling interpersonal relationships and place-mediated interactions may improve transmission inference when relevant contact pathways are not fully captured by social network data alone.

While our Bayesian framework demonstrates strong performance in reconstructing transmission networks and identifying direct transmission pairs, several limitations merit consideration. First, limited availability of pathogen genome sequences constrains the model’s ability to accurately estimate key parameters, including infection rate, mutation rate (µ), and within-host effective population size (θ). In smaller datasets, the predominance of indirect transmissions can inflate estimates of µ and θ, reducing the sensitivity of hypothesis testing for direct transmission detection. Additionally, the framework assumes a static contact network, even though real-world social and spatial connections often evolve over the course of an outbreak. Although incorporating a time-varying network would better capture these dynamic interactions, it would substantially increase the complexity of inferring transmission events. Moreover, the assumption of a uniform within-host effective population size across hosts oversimplifies biological reality; relaxing this assumption would increase model complexity and require substantially larger datasets for reliable inference. Future improvements could integrate richer data sources and refine model assumptions, for example, incorporating mobility data or transient encounters predicted from GPS or mobile trajectories using machine learning. Such enhancements would enable more precise modeling of contact likelihood and improve the accuracy of transmission inference.

The computational demands of Bayesian inference in large-scale datasets remain a practical challenge. For example, analyzing real-world data comprising 93 tuberculosis cases required a few minutes on a standard desktop. As the number of cases grows, computation time can increase exponentially due to the complexity of Markov Chain Monte Carlo (MCMC) sampling and likelihood evaluations. Scaling this framework for larger outbreaks or population-level studies will require algorithmic optimization and parallel computing strategies. Leveraging high-performance computing resources and exploring more efficient sampling techniques, such as Hamiltonian Monte Carlo or variational inference, could significantly reduce runtime and enable broader epidemiological applications. These improvements are essential for translating network-informed Bayesian models into real-time public health decision-making tools.

## Supplementary Information

Below is the link to the electronic supplementary material.

Supplementary Material 1

## Data Availability

The network-informed Bayesian model has been implemented in Julia and is available at https://github.com/lliu1871/betnet.
