# Supplementary material for: A Bayesian Framework for the Network Analysis of Transmission Dynamics in Infectious Disease
Source: J Mol Evol. 2026 Feb 18;94(2):316–27. doi: 10.1007/s00239-026-10303-w (PMC13076497; doi:10.1007/s00239-026-10303-w)

Supplementary Materials

Jianing Xu, Jihyun Kim, Pengsheng Ji, Lili Yu, Christopher C. Whalen, Liang Liu

**S1: Hypothesis Testing of Direct Transmissions using L-BFGS-B optimization**

We employ a limited-memory BFGS algorithm with box constraints (L-BFGS-B) (Byrd et al. 1995) to re-estimate $\theta$ and $\mu$ from direct transmission pairs. L-BFGS-B is efficient, stable, and accommodates bound constraints, making it well suited for likelihood-based inference. By optimizing parameters in a data-driven manner, the test gains sensitivity in detecting direct transmission. Although L-BFGS-B supports bound constraints, setting very wide (uninformative) bounds can inflate type I error. Hence, we restrict estimation to a region around posterior means:

$$L_{\alpha}=\max\left( 0, \hat{\alpha}- \lambda\hat{\sigma_{\alpha}} \right), U_{\alpha} = \hat{\alpha}+ \lambda\hat{\sigma_{\alpha}}$$

where $\hat{\alpha}$ and $\hat{\sigma_{\alpha}}$ are the posterior mean and standard deviation of the parameter $\alpha=\theta,\mu$. The tuning constant $\lambda$ determines the range: $\lambda=0$ reduces to the posterior-only method (no optimization), while $\lambda\to\infty$ approximates unrestricted optimization. Thus, $\lambda$ provides a continuum between conservative and flexible estimation, enabling sensitivity analysis of the trade-off between type I control and power.

The optimization algorithm begins by using posterior estimates of the effective population size (θ) and mutation rate (μ), along with their standard deviations, to define initial bounds for parameter optimization (Algorithm1). These bounds are controlled by a predefined tuning constant λ, which determines how far the optimization can deviate from the posterior means. The optimization of θ and μ involve two steps. In the first step, the algorithm minimizes the negative log-likelihood of observed SNP differences among direct transmission pairs to obtain refined estimates of θ and μ. This step is implemented in R using the *optim* function with the “L-BFGS-B” method, which applies the trusted-region bounds to perform constrained minimization of the negative log-likelihood. In the second step, these parameters are used to update the hypothesis testing threshold and reclassify transmission pairs. This process is repeated until the classification stabilizes, meaning no further changes occur in the assignment of direct or indirect transmission links. The final output includes the optimized parameter values and the updated classification of transmission pairs, enhancing the sensitivity of the hypothesis test while maintaining control over type I error.

| **Algorithm 1: Optimization of** $\boldsymbol{\theta}$ **and** $\boldsymbol{\mu}$ **for Transmission Pair Classification** | | |
| --- | --- | --- |
|  | Use Bayesian estimates of $\theta\left( \hat{\theta},\hat{\sigma}_{\theta} \right),$ and $\mu\left( \hat{\mu},\hat{\sigma}_{\mu} \right)$ to establish an initial threshold and determine direct/indirect transmission pairs with hypothesis testing. | |
|  | **Repeat** | |
|  |  | Under preselected $\lambda$, optimize $\theta$ and $\mu$ by minimizing the negative loglikelihood of SNP differences given time for $k$ direct pairs.  Define the objective function as:  $-\sum_{i=1}^{k} log\left[ P\left( {SNP}_{1} \right\vert time_{1} , \theta, \mu\right)]$  Set bounds for $\theta$ and $\mu$ as:  $L_{\theta} =\max\left( 0, \hat{\theta}- \lambda\hat{\sigma}_{\theta} \right), U_{p} = \hat{\theta}+ \lambda\hat{\sigma}_{\theta}$  $L_{\mu} =\max\left( 0, \hat{\mu}- \lambda\hat{\sigma}_{\mu} \right), U_{p} = \hat{\mu}+ \lambda\hat{\sigma}_{\mu}$  Apply the L-BFGS-B to estimate optimal values $\theta^{*} , \mu^{*}$.  Update the threshold and reclassify transmission pairs. |
|  | **Until** | No further changes in classification of transmission pairs. |
|  | **Output** | Optimized parameter values $\theta^{*} , \mu^{*}$, and final classification of pairs. |

To select the optimal tuning parameter λ, the algorithm (Algorithm2) begins by defining a set of candidates λ and applying the optimization algorithm to each of three replicate datasets under the same simulation scenario. For each replicate, the algorithm iteratively tests increasing λ values, optimizing model parameters and evaluating hypothesis test performance until the type I error exceeds 5%. Among the feasible λ values (those maintaining type I error below the threshold), the one that maximizes the power of the hypothesis test is selected for each replicate. The minimum of these three replicate-specific λ values is then designated as the conservative scenario-level λ_min_. Finally, each replicate is re-evaluated using λ_min_ to confirm type I error control and assess power, yielding a robust and generalizable selection for λ across similar modeling scenarios.

| **Algorithm 2:** $\boldsymbol{\lambda}$ **Selection and Scenario-Level Guidance** | | |
| --- | --- | --- |
|  | Use ordered candidate value $\Lambda=\left\{ \lambda_{1}<\lambda_{2}<\cdot\cdot\cdot<\lambda_{k} \right\};$three replicate data under same scenario (parameter setting and sample size), with Bayesian estimates of $\theta\left( \hat{\theta},\hat{\sigma}_{\theta} \right),$ and $\mu\left( \hat{\mu},\hat{\sigma}_{\mu} \right)$. | |
|  | **Repeat for each replicate**  Start with the smallest value $\lambda_{1}$  **Repeat**  Run Algorithm 1 with current $\lambda_{i},$ obtaining optimal $\theta$ and $\mu$.  Perform hypothesis testing under the optimal values.  Record type I error and power  Move to the next $\lambda_{i+1}$  **Until** the next candidate would cause type I error to exceed $5\%$  **Then** among feasible $\left\{ \lambda_{1},\lambda_{2},\cdot,\lambda_{i} \right\}$, select $\lambda_{r}$ that maximize power | |
|  |  |  |
|  | **Until**  **Then** | all three replicates have their replicate-specific choice $\lambda_{r}$.  Define the conservative scenario-level guidance as  $\lambda_{min}=min(\lambda_{r1},\lambda_{r2},\lambda_{r3}).$ |
|  | Re-evaluate each replicate at $\lambda_{min}$​, recording type I error and power. | |
|  | **Output:** Replicate-specific choices $\lambda_{r}$​, scenario-level $\lambda_{min}$​; and associated type I error and power. | |

**S2: Non-network-based and network-based simulation**

Network-based simulation: we construct a contact proximity network $\Psi$ of 10,000 nodes with a hybrid approach that combines the Erdős-Rényi (ER) and Barabási-Albert (BA) models, both implemented in the igraph package in R. The ER model generates a sparse core of 1,000 nodes with a low edge probability (p = 0.002), capturing limited initial connectivity. This core is expanded to 10,000 nodes using the BA model with preferential attachment (m = 1), producing a heterogeneous degree distribution with central hubs. This structure reflects the variability in social connectivity observed in real-world contact networks and serves as the foundation for simulating disease transmission. All edges in the network are assigned unit length, so that geodesic distance reflects the number of social steps between individuals, serving as a meaningful proxy for contact proximity and transmission likelihood. Based on this contact proximity network Ψ, we simulate disease spread over time by initiating infection in a randomly selected individual and iteratively modeling transmission events across the network. We assume that each individual can be infected at most once, and that transmission events occur independently between infectious and susceptible individuals. The first infectious individual is randomly selected from the 10,000 individuals in the network $\Psi$. We set his/her infection time to $T_{1}^{I}=0$. The onset time $T_{1}^{O}$ is then sampled from a scaled chi-squared distribution $\frac{1}{14}\cdot\chi_{8}^{2}$, and the removal time $T_{1}^{R}$ s drawn from an exponential distribution with rate $\beta$. This individual $a$ becomes the initial source of transmission. All susceptible individuals connected to $a$ in $\Psi$ are identified, forming potential transmission pairs $a\to b_{1},\ldots,a\to b_{m_{1}}$. For each pair $\left( a,b \right)$, a transmission event is simulated as a Bernoulli trial with probability:

$$p_{a,b}=c_{a,b}\cdot k_{a}$$

where $c_{a,b}=e^{-d_{a,b}}$ represents the contact probability between $a$ and $b$ based on network distance $d_{a,b}$ in $\Psi$, and $k_{a}$ is the infectiousness of individual $a$ over his/ her infectious period, defined as:

$$k_{a}=P\left( t_{b}^{I}\leq T_{a}^{R} - T_{a}^{O} \right)=1-e^{-\alpha\left( T_{a}^{R} - T_{a}^{O} \right)}$$

with $\alpha$ representing the infection rate. If transmission occurs, the infection time of recipient $b$ is modeled as the delay between the onset of infectiousness in *a* and transmission to $b$, drawn from an exponential distribution with rate $\lambda$. However, transmission can only occur if this delay falls within the infectious period of *a*, i.e., $t_{b}^{I}=T_{b}^{I}-T_{a}^{O}\leq T_{a}^{R} - T_{a}^{O}$​. Accordingly, the infection delay follows a truncated exponential distribution with upper bound $T_{a}^{R} - T_{a}^{O}$​. After determining the infection time, the onset and removal times for individual $b$ are generated using the same distributions as for the index case. Among all newly infected individuals, the one with the earliest infection time is selected as the next transmission source. This process is repeated iteratively: identifying new susceptible neighbors, simulating transmission events, and generating infection timelines, until a predefined stopping criterion is met. In our simulation, we terminate the process once 500 individuals have been infected. This framework captures the temporal dynamics of transmission, incorporates variable infectious periods, and accounts for heterogeneous contact probabilities based on network structure.

**S3: Simulating SNP counts from direct and indirect transmission pairs**

1. Direct Transmission Pairs

For a direct transmission pair $(j\to i)$, where individual $j$ is the Bayesian-estimated transmitter of $i$,the number of SNPs - i.e., the number of base differences between two genomes of $i$ and $j$ - is sampled from a Binomial distribution with parameters $N$ and $p_{i,j}$, where $N$ is the genome length and $p_{i,j}$ is the probability of mutation between the genomes of individuals $i$ and $j$. The mutation probability $p_{i,j}$ is calculated as

$$p_{i,j} = \frac{3}{4} - \frac{3}{8\theta+ 4} e^{-\mu(t_{i,1} + t_{i,2})}$$

where $\theta$ and $\mu$ are the effective population size and mutation rate, respectively. The terms $t_{i,1}$ and $t_{i,2}$ represent the time intervals $T_{i}^{R}-T_{i}^{I}$ and $T_{j}^{R}-T_{i}^{I}$. Each SNP is placed at a random locus, and the nucleotide substitution is randomly chosen from the three alternatives to the original base. Individual $i$’s genome is constructed by inheriting the set of mutations from transmitter $j$, with additional mutation introduced during the transmission event. If a newly acquired mutation occurs at a locus that already carries an inherited mutation, the new mutation overwrites the inherited one. As a result, individual $i$’s genome is represented as a modified version of $j$’s, capturing both inherited and updated mutation information relative to the reference genome.

1. Indirect Transmission Pairs

For indirect transmission pairs, the SNP count is determined by combining all mutation loci found in either individual’s sequence — this includes both inherited mutations from intermediate hosts and independently acquired mutations. For example, when comparing patient 3 to the reference genome of patient 1, patient 3’s genome includes the inherited mutation at locus 2 and an additional mutation at locus 4, resulting in a total of two SNPs. By recording only mutation loci instead of complete genomes, this method efficiently captures genomic variation while significantly reducing storage demands.

**S4: Combinations of μ and θ evaluated under** $\boldsymbol{N}\boldsymbol{=4.4\times}\mathbf{10}^{\mathbf{6}}$**, with** $\boldsymbol{\alpha}\mathbf{=}\boldsymbol{\beta}\mathbf{=3}$**. The red X indicates combinations that were also examined under** $\boldsymbol{\alpha}\mathbf{=}\boldsymbol{\beta}\mathbf{=2}$ **and** $\boldsymbol{\alpha}\mathbf{=}\boldsymbol{\beta}\mathbf{=1.5}$**. Parentheses indicate the scenario numbers corresponding to each case. For example, the cell labeled *X (S1, S6, S7)* represents the same μ and θ values evaluated in Scenario 1 (α = β = 3), Scenario 6 (α = β = 2), and Scenario 7 (α = β = 1.5).**

| $\boldsymbol{\mu\backslash\theta}$ | $\boldsymbol{1\times}\boldsymbol{10}^{\boldsymbol{-6}}$ | $\boldsymbol{2\times}\boldsymbol{10}^{\boldsymbol{-6}}$ | $\boldsymbol{5\times}\boldsymbol{10}^{\boldsymbol{-6}}$ |
| --- | --- | --- | --- |
| $\boldsymbol{5\times}\boldsymbol{10}^{\boldsymbol{-7}}$ | X (S1, S6, S7) | X (S6) | X (S3) |
| $\boldsymbol{1\times}\boldsymbol{10}^{\boldsymbol{-6}}$ | X (S2) |  | X (S4) |
| $\boldsymbol{2\times}\boldsymbol{10}^{\boldsymbol{-6}}$ | X (S5) |  |  |

**S5: Combinations of μ and θ evaluated under** $\boldsymbol{N=1\times}\boldsymbol{10}^{\boldsymbol{6}}$**, with** $\boldsymbol{\alpha=\beta=3}$**.**

| $\boldsymbol{\mu\backslash\theta}$ | $\boldsymbol{1\times}\boldsymbol{10}^{\boldsymbol{-6}}$ | $\boldsymbol{5\times}\boldsymbol{10}^{\boldsymbol{-6}}$ | $\boldsymbol{1\times}\boldsymbol{10}^{\boldsymbol{-5}}$ |
| --- | --- | --- | --- |
| $\boldsymbol{5\times}\boldsymbol{10}^{\boldsymbol{-7}}$ | X | X |  |
| $\boldsymbol{1\times}\boldsymbol{10}^{\boldsymbol{-6}}$ | X | X | X |
| $\boldsymbol{5\times}\boldsymbol{10}^{\boldsymbol{-6}}$ |  |  | X |

**S6: The algorithm for network perturbation and ERGM fitting**

To evaluate the robustness of transmission network inference under structural uncertainty, this algorithm introduces systematic perturbations to a simulated network of 10,000 nodes. For each noise level (5%, 10%, and 20%), a predefined proportion of node pairs is randomly selected, and their connections are either removed or added to simulate network noise. This perturbation process is repeated across three iterations per noise level. Following each perturbation, network distances among individuals in the transmission subset are recalculated, and an Exponential Random Graph Model (ERGM) is fitted using the updated adjacency matrix. This approach enables quantification of the impact of network noise on transmission inference, offering insights into the stability and reliability of ERGM-based modeling under varying degrees of data perturbation.

| **Algorithm 3: Network Perturbation and ERGM Fitting** | | | | | |
| --- | --- | --- | --- | --- | --- |
|  | Retrieve the adjacency matrix from the previously simulated network of  $10,000$ nodes. | | | | |
|  | **for** each noise level ($5\%, 10\%, and 20\%$) **do** | | | | |
|  |  | **for** iteration = 1 to 3 **do** | | | |
|  |  | | Select a predefined percentage of node pairs (e.g., $10\%$). | | |
|  |  | | **for** each selected node pair $(i, j)$ **do** | | |
|  |  | | | **if** adjacency matrix entry $A[i, j] = 1$ **then** | |
|  |  | | | | Set $A\left[ i, j \right]\to0$ (remove tie). |
|  | | | | **else if** $A[i, j] = 0$ **then** | |
|  | | | | | Set $A\left[ i, j \right]\to1$ (add tie). |
|  | | | | **end if** | |
|  | | | **end for** | | |
|  | | | Recompute network distances for individuals in the transmission  network (a subset of the full $10,000$-node network) based on the modified adjacency matrix. | | |
|  | | | Fit the ERGM using the transmission tree, incorporating the up-  dated network distance matrix. | | |
|  | | **end for** | | | |
|  | **end for** | | | | |

S7: The mean power of the hypothesis tests under different parameter variations across sample sizes. a) The mean power for varying θ values across sample sizes, with μ fixed at $5\times{10}^{-7}$ and α = β = 3. b) The mean power for varying μ values across sample sizes, with θ fixed at $1\times{10}^{-6}$and α = β = 3. c) The mean power for varying α and β across sample sizes, with θ fixed at $1\times{10}^{-6}$and μ fixed at $5\times{10}^{-7}$.


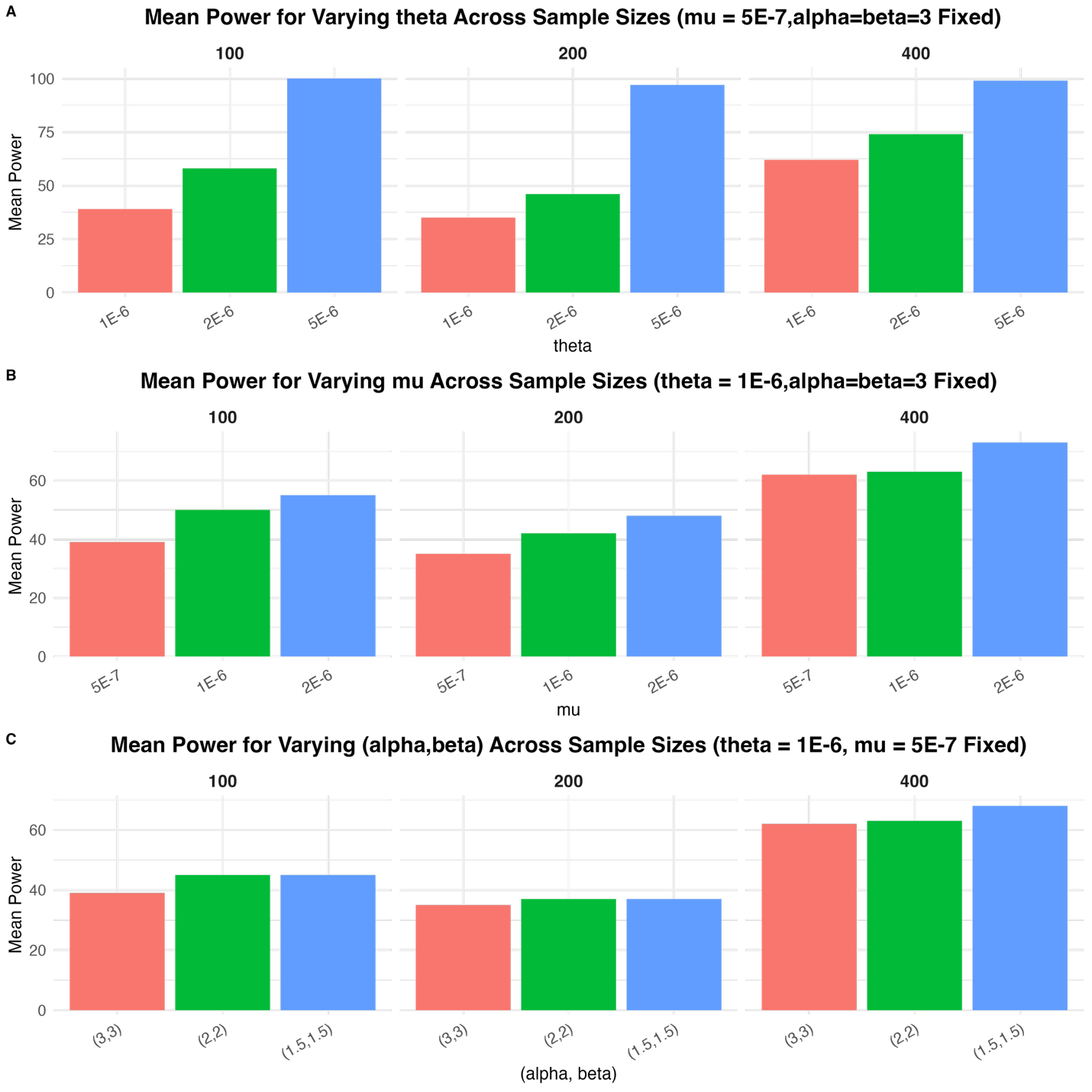


**S8: Posterior Estimates of Parameters**

|  | Posterior Estimates of Merged Runs: Mean; $95\%$ Credible Interval | |
| --- | --- | --- |
|  | Bayesian without Network | Bayesian with Network |
| $\alpha$ | $2.74; [2.21,3.33]$ | $2.74; [2.21,3.32]$ |
| $\theta$ | $8.70\times{10}^{-7};$  $[4.45\times{10}^{-7},1.28\times{10}^{-6}]$ | $2.50\times{10}^{-7};$  $[1.05\times{10}^{-8}, 6.97\times{10}^{-7}]$ |
| $\mu$ | $2.36\times{10}^{-6};$  $[1.62\times{10}^{-7}, 3.23\times{10}^{-6}]$ | $3.06\times{10}^{-6};$  $[2.39\times{10}^{-7}, 3.63\times{10}^{-6}]$ |

**S9: Identification of 27 direct transmissions in the estimated transmission network of 93 strains under the Bayesian Model with Temporal and Genomic Data; 13 Highlighted rows are the pairs are the ones identified as direct in both models.**

| Case ID | Case ID (Transmitter) | Upper Bound C | # of SNPs | Network Distance |
| --- | --- | --- | --- | --- |
| 17 | 6 | 21.14 | 8 | 9 |
| 18 | 16 | 15.85 | 1 | Inf |
| 24 | 3 | 21.87 | 1 | 8 |
| 25 | 20 | 28.48 | 27 | Inf |
| 26 | 16 | 19.75 | 1 | 9 |
| 27 | 2 | 23.48 | 1 | 9 |
| 28 | 17 | 18.90 | 10 | Inf |
| 30 | 19 | 21.28 | 4 | 9 |
| 32 | 29 | 14.92 | 3 | Inf |
| 33 | 19 | 17.23 | 0 | Inf |
| 37 | 30 | 22.30 | 0 | 7 |
| 49 | 33 | 17.99 | 0 | Inf |
| 50 | 35 | 15.59 | 4 | 13 |
| 52 | 28 | 16.66 | 1 | Inf |
| 53 | 26 | 20.77 | 2 | Inf |
| 54 | 44 | 15.20 | 0 | Inf |
| 58 | 17 | 23.56 | 13 | 6 |
| 59 | 13 | 20.12 | 1 | 11 |
| 62 | 49 | 16.75 | 3 | 2 |
| 66 | 39 | 15.37 | 15 | Inf |
| 67 | 63 | 17.20 | 17 | Inf |
| 72 | 53 | 17.26 | 10 | Inf |
| 78 | 36 | 27.39 | 8 | 10 |
| 79 | 39 | 29.21 | 17 | 6 |
| 83 | 69 | 19.13 | 0 | 15 |
| 86 | 53 | 24.41 | 13 | Inf |
| 87 | 45 | 29.66 | 20 | 9 |

**S10: Identification of 17 direct transmissions in the estimated transmission network of 93 strains under the Bayesian Model with Temporal Genomic and Network Data; 13 Highlighted rows are the pairs are the ones identified as direct in both models.**

| Case ID | Case ID (Transmitter) | Upper Bound C | # of SNPs | Network Distance |
| --- | --- | --- | --- | --- |
| 17 | 6 | 21.42 | 8 | 9 |
| 24 | 3 | 29.67 | 1 | 8 |
| 26 | 18 | 10.16 | 4 | 10 |
| 27 | 2 | 21.90 | 1 | 9 |
| 30 | 19 | 18.40 | 4 | 9 |
| 37 | 30 | 18.34 | 0 | 7 |
| 49 | 37 | 12.49 | 1 | 10 |
| 50 | 35 | 10.63 | 4 | 13 |
| 58 | 17 | 22.27 | 13 | 6 |
| 59 | 13 | 26.71 | 1 | 11 |
| 62 | 49 | 11.32 | 3 | 2 |
| 72 | 26 | 20.09 | 17 | 13 |
| 78 | 36 | 24.35 | 8 | 10 |
| 79 | 39 | 25.49 | 17 | 6 |
| 83 | 69 | 15.53 | 0 | 15 |
| 85 | 20 | 38.70 | 37 | 15 |
| 87 | 45 | 28.66 | 20 | 9 |

**S11: ERGM Summary of Inferred Transmission Network under Bayesian Model with Temporal and Genomic Data**


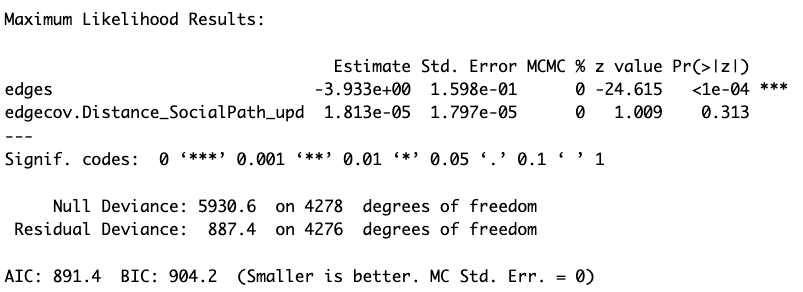


**S12: ERGM Summary of Inferred Transmission Network under Bayesian Model with Temporal, Genomic and Network Data**


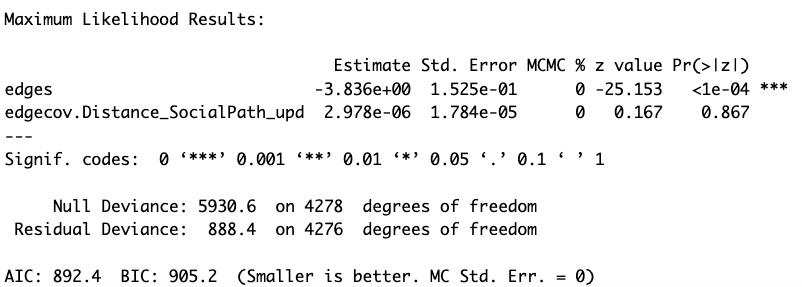


**S13: The non–network-informed Bayesian model infers transmission events exclusively based on pairwise SNP distances (black edges), whereas the network-informed Bayesian model integrates both genetic (SNP) distances and network-based proximity (yellow edges) to infer transmission pathways. Solid yellow edges represent direct connections between nodes within the network, while dashed yellow edges indicate the absence of such connections. The yellow node identifies the inferred infector of the green node under the network-informed model, whereas the black node denotes the inferred infector under the non–network-informed model.**


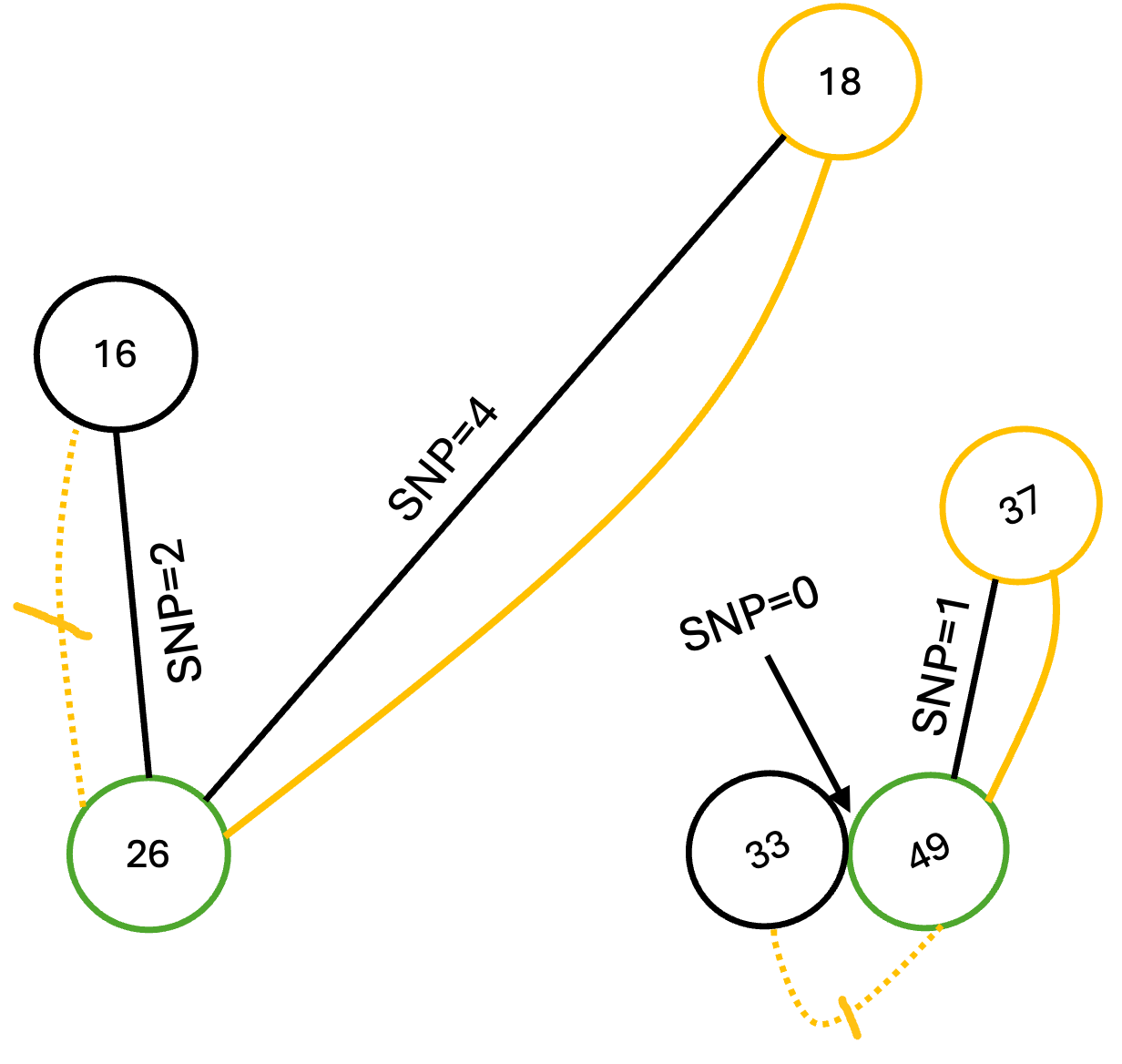

Supplement: Supplementary file 1 — Supplementary Material 1 [file 239_2026_10303_MOESM1_ESM.docx]
